# A Distinct Nasal Microbiota Signature in Peritoneal Dialysis Patients

**DOI:** 10.34067/KID.0000000000000249

**Published:** 2023-08-29

**Authors:** Iman Khan, Sylvia Wu, Anika Hudson, Clayton Hughes, Gabriel Stryjniak, Lars F. Westblade, Michael J. Satlin, Nicholas Tedrow, Anne-Catrin Uhlemann, Colleen Kraft, Darshana M. Dadhania, Jeffrey Silberzweig, Iwijn De Vlaminck, Carol Li, Vesh Srivatana, John Richard Lee

**Affiliations:** 1 Division of Nephrology and Hypertension, Department of Medicine, Weill Cornell Medicine, New York, New York; 2 Department of Pathology and Laboratory Medicine, Weill Cornell Medicine, New York, New York; 3 Division of Infectious Diseases, Department of Medicine, Weill Cornell Medicine, New York, New York; 4 Division of Infectious Diseases, Department of Medicine, Vagelos College of Physicians and Surgeons Columbia University, New York, New York; 5 Division of Infectious Diseases, Department of Medicine, Emory University School of Medicine, New York, New York; 6 Department of Transplantation Medicine, New York Presbyterian Hospital–Weill Cornell Medical Center, New York, New York; 7 The Rogosin Institute, New York, New York; 8 Nancy E. and Peter C. Meinig School of Biomedical Engineering, Cornell University, Ithaca, New York

**Keywords:** peritoneal dialysis, transplantation

## Abstract

**Key Points:**

- *Staphylococcus*, *Corynebacterium*, *Streptococcus*, and *Anaerococcus* are the most common genera in the anterior nares.
- The nasal abundance of *Staphylococcus* is inversely correlated with the nasal abundance of *Corynebacterium*.
- Peritoneal dialysis patients have a distinctly diverse representation of *Staphylococcus* and *Streptococcus* in their anterior nares.

**Background:**

The nasal passages harbor both commensal and pathogenic bacteria that can be associated with infectious complications. The nasal microbiome in peritoneal dialysis (PD) patients, however, has not been well characterized. In this study, we sought to characterize the anterior nasal microbiota in PD patients and assess its association with PD peritonitis.

**Methods:**

In this study, we recruited 32 PD patients, 37 kidney transplant (KTx) recipients, and 22 living donor/healthy control (HC) participants and collected their anterior nasal swabs at a single point in time. We followed the PD patients for future development of peritonitis. We performed 16S ribosomal RNA (rRNA) gene sequencing of the V4–V5 hypervariable region to determine the nasal microbiota. We compared nasal abundance of common genera among the three groups using Wilcoxon rank-sum test with Benjamini–Hochberg adjustment. DESeq2 was also used to compare the groups at the amplicon sequence variant levels.

**Results:**

In the entire cohort, the most abundant genera in the nasal microbiota included *Staphylococcus*, *Corynebacterium*, *Streptococcus*, and *Anaerococcus*. Correlational analyses revealed a significant inverse relationship between the nasal abundance of *Staphylococcus* and that of *Corynebacterium*. PD patients have a higher nasal abundance of *Streptococcus* than KTx recipients and HC participants. PD patients have a more diverse representation of *Staphylococcus* and *Streptococcus* than KTx recipients and HC participants. PD patients who concurrently have or who developed future *Staphylococcus* peritonitis had a numerically higher nasal abundance of *Staphylococcus* than PD patients who did not develop *Staphylococcus* peritonitis.

**Conclusions:**

We find a distinct nasal microbiota signature in PD patients compared with KTx recipients and HC participants. Given the potential relationship between the nasal pathogenic bacteria and infectious complications, further studies are needed to define the nasal microbiota associated with these infectious complications and to conduct studies on the manipulation of the nasal microbiota to prevent such complications.

## Graphical abstract

**Figure:**
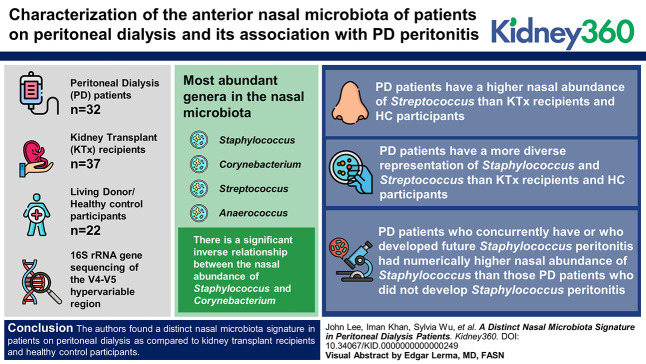

## Introduction

The anterior nasal microbiota is at the interface between the external environment and the nasal passages and contains a combination of commensal and pathogenic bacteria. The most common genera defined in healthy individuals in the Human Microbiome Project are *Staphylococcus*, *Corynebacterium*, *Propionibacterium*, and *Moraxella*.^1^ Subsequent studies on the nasal microbiota have revealed microbiota dysbiosis in diseased states, such as chronic rhinosinusitis,^2^ and have linked the nasal microbiota to infectious complications after elective surgical procedures.^3^

Peritoneal dialysis (PD) patients undergo dialysis through PD catheter through their abdomen. Despite being taught sterile technique, PD patients experience both exit-site infections around the catheter and infectious peritonitis. Prior work has established that pathogenic bacteria in the nasal passages may be associated with infectious complications in PD patients. Luzar *et al.* reported that *Staphylococcus aureus* (*S. aureus*) nasal colonization was associated with exit-site infections in a cohort of 140 PD patients.^4^ Other studies have found that persistent nasal colonization with *S. aureus* was also associated with peritonitis.^5,6^ Decolonization with mupirocin has been suggested to prevent infections, and the MUPIROCIN Study Group found that nasal mupirocin prevented *S. aureus* exit-site infection.^7^ As such, the International Society of PD Catheter-Related Infection Recommendation 2017 Update recommends screening for *S. aureus* and treating with intranasal mupirocin if present.^8^ No study, to date, has comprehensively evaluated the anterior nasal microbiota in PD patients, which could be useful to understand how mupirocin could be beneficial.

In this pilot study, we evaluated the anterior nasal microbiota using 16S rRNA gene sequencing of the V4–V5 hypervariable region in PD patients, kidney transplant (KTx) recipients, and healthy controls (HCs).

## Methods

### Study Cohort Recruitment and Nasal Swab Specimen Collection

From August 2021 to January 2022, we recruited patients receiving PD, KTx recipients, and living donor/HC participants for anterior nasal swab specimen collection. All KTx recipients and living donor candidates were recruited from the clinic. Most PD patients were recruited in the PD clinic; several were recruited during hospitalization. The Weill Cornell Institutional Review Board approved this protocol (1604017181), and all participants provided written informed consent. This study adhered to the principles of the Declaration of Helsinki. The clinical and research activities being reported are consistent with the Principles of the Declaration of Istanbul as outlined in the “Declaration of Istanbul on Organ Trafficking and Transplant Tourism.”

Anterior nasal swab specimens were collected once from each participant using the Human Microbiome Project protocol. A Copan Eswab (Copan Diagnostics, Murietta, CA) was inserted into the anterior part of one nostril of the participant and turned twice and was then inserted into the anterior part of the other nostril and turned twice. The Copan Eswab was then placed into 1 ml of liquid Amies provided by the Copan Eswab technology and immediately stored on ice or 4°C. Aliquots of 300 *µ*l were created in 2 ml cryovial and stored at −80°C within 12 hours.

### 16S rRNA Gene Sequencing of the V4–V5 Hypervariable Region

A single aliquot of approximately 285 *μ*l was deposited into a Qiagen PowerBead glass 0.1-mm tube. Using a Promega Maxwell RSC PureFood GMO and Authentication Kit (AS1600), 1ml of CTAB buffer and 20 *μ*l of RNAse A Solution were added to the PowerBead tube containing the sample. The sample/buffer was mixed for 10 seconds on a Vortex Genie2 and then incubated at 95°C for 5 minutes on an Eppendorf ThermoMixer F2.0, shaking at 1500 rpm. The tube was removed and clipped to a horizontal microtube attachment on a Vortex Genie2 (SI-H524) and vortexed at high speed for 20 minutes. The sample was removed from the Vortex and centrifuged on an Eppendorf Centrifuge 5430R at 40°C, 12,700 rpm for 10 minutes. On completion, the sample was centrifuged again for an additional 10 minutes to eliminate foam. The tube was then added to a Promega MaxPrep Liquid Handler tube rack. The Liquid Handler instrument was loaded with proteinase K tubes, lysis buffer, elution buffer, 1000 ml tips, 50 ml tips, 96-sample deep-well plate, and Promega Maxwell RSC 48 plunger tips. The Promega MaxPrep Liquid Handler instrument was programed to use 300 *μ*l of sample and transfer all sample lysate into Promega Maxwell RSC 48 extraction cartridge for DNA extraction. On completion, the extraction cartridge was loaded into Promega Maxwell RSC 48 for DNA extraction and elution. DNA was eluted in 100 *μ*l and transferred to a standard 96-well plate. DNA was quantified using Quant-iT double stranded DNA High-Sensitivity Assay Kit using Promega GloMax plate reader on a microplate (655087). 16S rRNA library generation followed the protocol from the Earth Microbiome Project.

Amplicon libraries were washed using Beckman Coulter AMPure XP magnetic beads. Library quality and size verification was performed using PerkinElmer LabChip GXII instrument with DNA 1K Reagent Kit (CLS760673). Library concentrations were quantified using Quant-iT double stranded DNA High-Sensitivity Assay Kit using Promega GloMax plate reader on a microplate (655087). Library molarity was calculated based on library peak size and concentration. Libraries were normalized to 2 nM using the PerkinElmer Zephyr G3 NGS Workstation (133750) and pooled together using the same volume across all normalized libraries into a 1.5 ml Eppendorf DNA tube (022431021). Sequencing was performed on an Illumina MiSeq instrument at loading concentration of 7 pM with 15% PhiX, paired-end 250 using MiSeq Reagent Kit v2, 500 cycles (MS-102-2003).

### Bioinformatics Pipeline

Demultiplexed raw reads were processed using the Nextflow^9^ nf-core^10^ ampliseq pipeline,^11^ version 2.2.0, with the following parameters: profile singularity—input SampleSheet.tsv—FW_primer GTGYCAGCMGCCGCGGTAA—RV_primer CCGYCAATTYMTTTRAGTTT—metadata Metadata.tsv—outdir results—dada_ref_taxonomy silva—ignore_empty_input_files—ignore_failed_trimming—min_frequency 10—retain_untrimmed—trunclenf 240—trunclenr 160. Specifically, reads were trimmed with cutadapt,^12^ PhiX and quality filtering, and read pair merging, and amplicon sequence variant (ASV) resolution was performed with DADA2.^13^ Subsequent taxonomic assignment was also performed with DADA2, using the Silva reference database,^14^ version 138. Sequences that were assigned the families, Chloroplast and Mitochondria, were removed from downstream analyses.

### Biostatistical Analyses

The distribution of categorical variables was compared using Fisher exact tests. The distribution of continuous variables was compared using Wilcoxon rank-sum tests, and to account for the comparison of multiple taxa, adjusted *P* values were calculated using Benjamini–Hochberg adjustment for multiple comparisons. DESeq2 was used to detect differences at the ASV between the groups using Benjamini–Hochberg adjustment. Comparison of correlations using a correlational matrix was adjusted for multiple comparisons using the Bonferroni method. All statistical tests were performed using R 4.1.3 in RStudio.

### Data Availability

Sequencing data that support the findings of this study will be made available in the database of Genotype and Phenotype phs002251.v1.p1 after peer-reviewed acceptance. Local institutional review board approval will be needed to access the data.

## Results

### Characteristics of the Study Cohort and Nasal Microbial Sequencing

The microbiota in the anterior nares was performed using 16S rRNA gene sequencing of the V4–V5 hypervariable region in 32 PD patients, 37 KTx recipients, 22 HC participants, and three negative controls. A total of 1,116,291 reads with assigned taxonomy were obtained in the cohort of 91 participants with a median of 12,713 assigned reads with an interquartile range of 7132 and 16,018 assigned reads. The number of assigned reads in the three negative controls was 146, 308, and 529, below the number in the cohort of participants.

Table 1 presents the demographics of the participants. In general, the PD patients were older than the HC participants and similar in age to the KTx recipients. More than 50% of PD patients performed automated PD, and 15% had current *Staphylococcus* peritonitis or developed future *Staphylococcus* peritonitis within 10–12 months from the nasal specimen collection (last follow-up). Approximately one third of KTx recipients received deceased donor transplantation, and 32% were on trimethoprim/sulfamethoxazole (TMP-SMX) prophylaxis.

**Table 1:** Demographics of the cohort

Characteristic	PD Cohort (*n*=32)	KTx Cohort (*n*=37)	HC Cohort (*n*=22)
Age, yr	63 (51–73)	59 (52–67)	51 (33–61)
Female sex	20 (63%)	17 (46%)	13 (59%)
**Ethnicity, *n* (%)**			
Hispanic	3 (9)	2 (5)	3 (14)
Non-Hispanic	27 (84)	33 (89)	16 (73)
Declined	2 (6)	2 (5)	3 (14)
**Race, *n* (%)**			
Asian	4 (11)	4 (11)	1 (5)
Black	13 (41)	10 (27)	5 (23)
White	11 (34)	20 (54)	11 (50)
Other	3 (9)	2 (5)	1 (5)
Declined	1 (3)	1 (3)	4 (18)
History of hypertension, *n* (%)	26 (81)	36 (97)	2 (9)
History of diabetes mellitus, *n* (%)	9 (28)	12 (32)	0 (0)
Years on PD	1.2 (0.6–2.4)		
Automated PD, *n* (%)	21 (66)		
**Concurrent or develops future, *n* (%)**			
*Staphylococcus* peritonitis	6 (19)		
Decreased donor transplantation		14 (38)	
Days post-transplantation		364 (51–1637)	
History of prior transplantation		4 (11)	
**Maintenance immunosuppression, *n* (%)**			
Tacro/mycophenolic mofetil		19 (51)	
Tacro/mycophenolic mofetil/pred		14 (38)	
Tacro/mycophenolic acid		1 (3)	
Tacro/mycophenolic acid/pred		3 (8)	
TMP-SMX PPx		12 (32)	

Categorical variables are represented by the number followed by the percentage in parentheses. Continuous variables are represented by the median followed by the interquartile range in parentheses. PD, peritoneal dialysis; KTx, kidney transplant; HC, healthy control; TMP-SMX, trimethoprim/sulfamethoxazole; ppx, prophylaxis; yr, year; Tacro, tacrolimus; pred, prednisone.

### Anterior Nasal Microbial Diversity Differs across the Study Cohort

**Figure 1:**
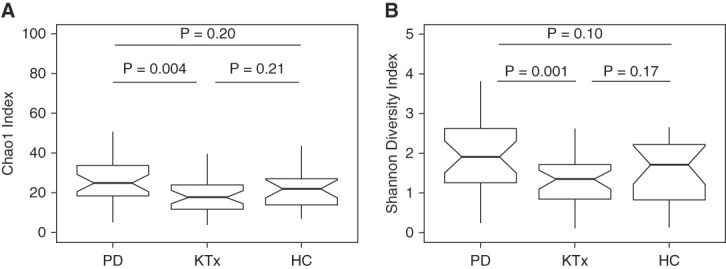
**Distinct differences in Nasal Microbial Diversity among the study cohort.** (A) Box and whisker plots of Chao1 index, the estimated number of ASVs, in the anterior nasal specimens from the PD cohort, the KTx cohort, and the living donor/HC cohort. The Chao1 index is on the *y* axis, and the study group is on the *x* axis. *P* value shown was calculated using the Wilcoxon rank-sum test. (B) Box and whisker plots of Shannon diversity index, a measure of evenness and richness, in the anterior nasal specimens from the three cohorts. The Shannon diversity index is on the *y* axis, and the study group is on the *x* axis. *P* values shown were calculated using the Wilcoxon rank-sum test. ASV, amplicon sequence variant; HC, healthy control; KTx, kidney transplant; PD, peritoneal dialysis.

Microbial diversity among the study participants was measured at the ASV level using the Shannon diversity, an index that evaluates the richness and evenness in a community, and Chao1, an index that estimates the total number of ASVs in the specimens. Figure 1, A and B, shows box and whisker plots of these diversity indices and reveals that PD patients had a significantly higher Shannon diversity index and Chao1 diversity index than KTx recipients (*P* < 0.05, Wilcoxon rank-sum test) but similar to the HC participants (*P* > 0.05).

### *Staphylococcus* Abundance Negatively Correlates with *Corynebacterium* Abundance in Anterior Nasal Specimens

**Figure 2:**
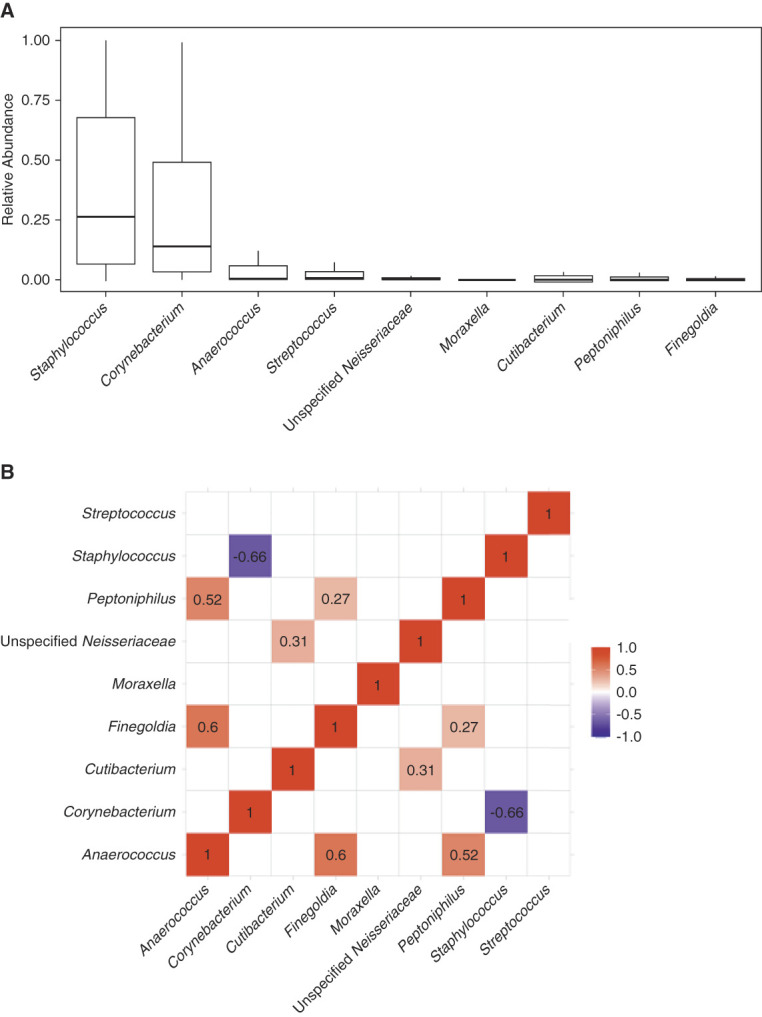
**Significant correlations among the most common genera in the study cohort.** (A) The most common genera in the anterior nasal microbiota (>1% mean relative abundance in the cohort). Box and whisker plots are represented to show the variation in the relative abundance of the genus (*y* axis) with the genus on the *x* axis. (B) A correlational matrix between the nasal abundance of the most common genera using Pearson *r* correlations with Benjamini–Hochberg adjustment for multiple hypotheses. The numbers shown are Pearson *r* correlations that had an adjusted *P* value <0.10. The color shows the strength of the correlation with red showing a positive correlation between two genera and blue showing a negative correlation between two genera.

We further evaluated the anterior nasal microbiota among the study cohort at the genus level. At the genus level, the top abundant genera (>1% mean abundance across the cohort) included *Staphylococcus*, *Corynebacterium*, *Anaerococcus*, *Streptococcus*, unspecified *Neisseriaceae*, *Moraxella*, *Cutibacterium*, *Peptoniphilus*, and *Finegoldia* (Figure 2A). We performed a correlational matrix analysis among each of the genera (Figure 2B). The relative abundance of *Staphylococcus* was inversely correlated with that of *Corynebacterium* (Pearson *r*=−0.66, adjusted *P* value <0.10, Benjamini–Hochberg adjustment). The relative abundance of *Peptoniphilus* was positively associated with that of *Anaerococcus* (*r*=0.52, adjusted *P* value <0.10) and *Finegoldia* (*r*=0.27, adjusted *P* value <0.10). The relative abundance of *Finegoldia* was positively associated with that of *Anaerococcus* (*r*=0.60, adjusted *P* value <0.10). The relative abundance of unspecified *Neisseriaceae* was positively associated with that of *Cutibacterium* (*r*=0.31, adjusted *P* value <0.10).

### Distinct Anterior Nasal Microbiota Define PD Patients and KTx Recipients

**Figure 3:**
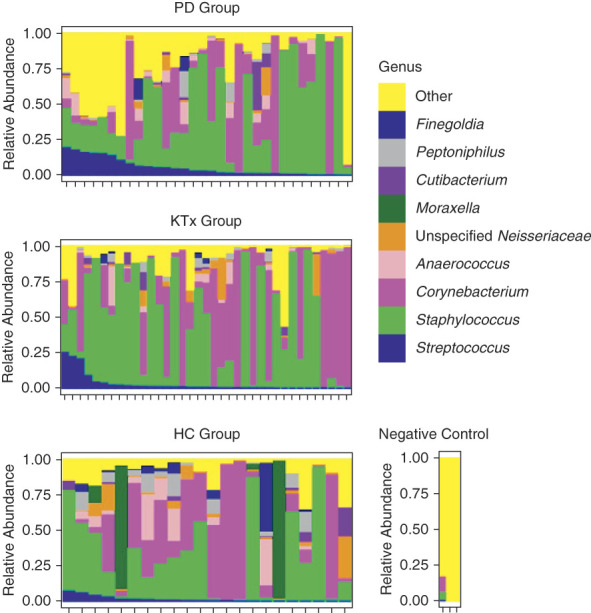
**Individual microbiota profiles by the study cohort.** The relative abundance of microbiota is on the *y* axis, and individual nasal specimens are on the *x* axis. The relative abundance of each genus is represented by color. The top panel represents anterior nasal microbiota profiles from the 32 PD patients, the middle panel represents the anterior nasal microbiota profiles from the 37 KTx patients, and the bottom panel represents the anterior nasal microbiota profiles from the 22 potential living donor/HC participants. The right panel represents the microbiota from three negative controls. HC, healthy control; KTx, kidney transplant; PD, peritoneal dialysis.

**Figure 4:**
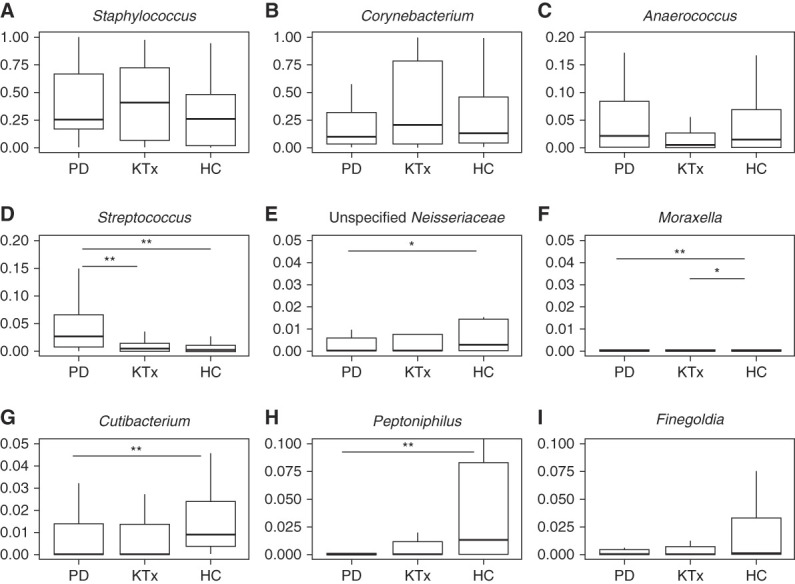
**Distinct microbial differences among the cohort at the genus level.** Box and whisker plots are represented with the relative abundance of individual genera on the *y* axis and the group on the *x* axis. PD cohort (*n*=32). KTx cohort (*n*=37). Living donor/HC cohort (*n*=22). *P* values were calculated using the Wilcoxon rank-sum test with Benjamini–Hochberg adjustment for multiple hypotheses. **Adjusted *P* value <0.05. *Adjusted *P* value <0.10. (A) *Staphylococcus* analysis. (B) *Corynebacterium* analysis. (C) *Anaerococcus* analysis. (D) *Streptococcus* analysis. (E) Unspecified *Neisseriaceae* analysis. (F) *Moraxella* analysis. (G) *Cutibacterium* analysis. (H) *Peptoniphilus* analysis. (I) *Finegoldia* analysis. HC, healthy control; KTx, kidney transplant; PD, peritoneal dialysis.

The individual profiles of the top genera in anterior nasal microbiota are shown in PD patients, KTx recipients, and HC participants (Figure 3). Figure 4 shows box and whisker plots of the top nine taxa among PD patients, KTx recipients, and HC participants, and Table 2 presents the comparisons among the groups using Wilcoxon rank-sum test with Benjamini–Hochberg adjustment. PD patients had a distinctly higher relative abundance of *Streptococcus* than KTx recipients or HC participants (adjusted *P* value <0.10, Wilcoxon rank-sum test, Benjamini–Hochberg adjustment) (Figure 4D). PD patients also had lower abundance of unspecified *Neisseriaceae*, *Moraxella*, *Cutibacterium*, and *Peptoniphilus* than HC participants (Figure 4G) (adjusted *P* value <0.10). KTx recipients had a lower abundance of *Moraxella* than HC participants (adjusted *P* value <0.10). Other than *Streptococcus*, KTx recipients had similar abundance of the top genera compared with PD patients (adjusted *P* value >0.10).

**Table 2:** Comparison of the nasal abundance among the three cohorts at the genus level

Genus	PD Cohort (*n*=32)	KTx Cohort (*n*=37)	HC Cohort (*n*=22)	PD versus HC	PD versus HC	PD versus KTx	PD versus KTx	KTx versus HC	KTx versus HC
	Median Abundance	Median Abundance	Median Abundance	*P* Value	Adjusted *P* Value	*P* Value	Adjusted *P* Value	*P* Value	Adjusted *P* Value
*Anaerococcus*	0.020	0.004	0.014	0.999	0.999	0.238	0.722	0.303	0.389
*Corynebacterium*	0.098	0.200	0.127	0.509	0.573	0.271	0.722	0.701	0.701
*Cutibacterium*	0.000	0.000	0.009	0.016^a^	0.049^a^	0.942	0.942	0.029	0.133
*Finegoldia*	0.000	0.000	0.001	0.098	0.147	0.660	0.880	0.178	0.320
*Moraxella*	0.000	0.000	0.000	0.014^a^	0.049^a^	NA	NA	0.008^a^	0.074^a^
Unspecified *Neisseriaceae*	0.000	0.000	0.003	0.034^a^	0.062^a^	0.510	0.817	0.105	0.237
*Peptoniphilus*	0.000	0.000	0.013	0.022	0.049^a^	0.382	0.763	0.046	0.139
*Staphylococcus*	0.249	0.403	0.257	0.406	0.522	0.891	0.942	0.285	0.389
*Streptococcus*	0.027	0.005	0.003	0.001^a^	0.008^a^	0.003^a^	0.027^a^	0.584	0.657

The median abundance of the most common genera is shown for the peritoneal dialysis cohort, the kidney transplant cohort, and the living donor/healthy control cohort. *P* values shown were calculated using the Wilcoxon rank-sum test between groups. Adjusted *P* values were calculated using Benjamini–Hochberg adjustment. *P* values with NA were unable to be calculated because the abundances were 0 in both groups. PD, peritoneal dialysis; KTx, kidney transplant; HC, healthy control; NA, not available.

a *P* value or adjusted *P* value <0.10.

**Figure 5:**
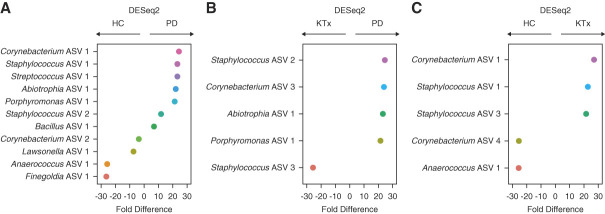
**Differential abundance analyses among the cohort at the ASV level.** Differential abundance analyses were performed on the anterior nasal microbiota between the groups using DESeq2 with Benjamini–Hochberg adjustment for multiple hypothesis testing. On the *y* axis is the individual ASV with genus shown, and on the *x* axis is the fold difference in abundance. The fold difference directionality is represented above the graph. (A) Differential abundance analyses between the HC group and the PD group. (B) Differential abundance analyses between the KTx group and the PD group. (C) Differential abundance analyses between the HC group and the KTx group. ASV, amplicon sequence variant; HC, healthy control; KTx, kidney transplant; PD, peritoneal dialysis.

To gain further insight, we evaluated the taxa at the ASV level. We performed pairwise DESeq2 between the groups to identify ASVs that were consistently different among the groups. Figure 5 shows the significant log2-fold abundance changes between the groups, and Supplemental Tables 1–3 reveal the changes in the nasal abundances of the groups. Both PD patients and KTx recipients had significantly higher nasal abundances of *Staphylococcus* ASV 1 and *Corynebacterium* ASV 1 and lower abundance of *Anaerococcus* ASV 1 than HC participants (adjusted *P* value <0.10, Benjamini–Hochberg adjustment). PD patients had higher nasal abundance of *Staphylococcus* ASV 2, *Abiotrophia* ASV 1, and *Porphyromonas* ASV 1 than KTx recipients or HC participants (adjusted *P* value <0.10).

### PD Patients Have a More Diverse Representation of Staphylococcus and Streptococcus than KTx Recipients and HC Participants

**Figure 6:**
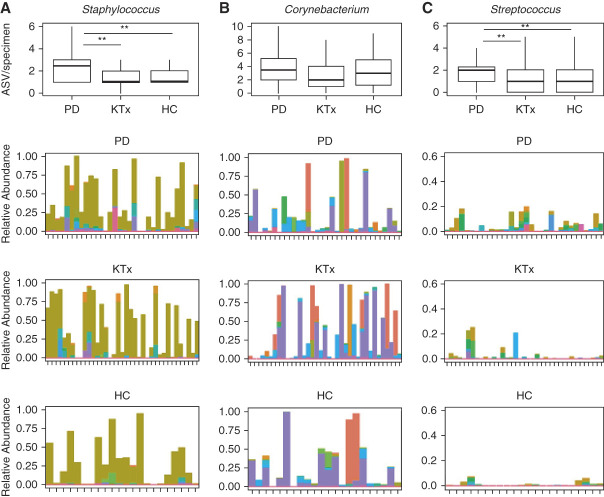
**Diverse representation of the most common genera in the study cohort.** Each set of graphs represents the number of ASV from a particular genus by the group. PD group (*n*=32). KTx group (*n*=37). Living donor/HC group (*n*=22). The top graph presents box and whisker plots of the number of ASVs per specimen by the group. *P* value was calculated using the Wilcoxon rank-sum test: **P value <0.05, *P value <0.10. The bottom three graphs represent box and whisker plots of the relative abundance of individual ASVs from a particular genus. The relative abundance is on the *y* axis with the color representing individual ASVs, and anterior nasal specimens are on the *x* axis with the second top graph representing the PD cohort, the second bottom graph representing the KTx cohort, and the bottom graph representing the HC cohort. (A) The diversity of *Staphylococcus* ASVs in the study cohort. (B) The diversity of *Corynebacterium* ASVs in the study cohort. (C) The diversity of *Streptococcus* ASVs in the study cohort. ASV, amplicon sequence variant; HC, healthy control; KTx, kidney transplant; PD, peritoneal dialysis.

To further understand why PD patients have higher microbial diversity, we evaluated the diversity of ASVs in the most common genera: *Staphylococcus*, *Corynebacterium*, and *Streptococcus* (Figure 6)*.* There were 61 different *Staphylococcus* ASVs identified in the whole cohort. PD patients had a significantly higher number of *Staphylococcus* ASVs per specimen than KTx patients (*P* = 0.03, Wilcoxon rank-sum test) and HC participants (*P* = 0.04) (Figure 6A). There were 95 different *Corynebacterium* ASVs identified in the whole cohort. PD patients, KTx patients, and HC participants had similar number of *Corynebacterium* ASVs per specimen (*P* > 0.10) (Figure 6B). There were 46 different *Streptococcus* ASVs identified in the whole cohort. PD patients had a significantly higher number of *Streptococcus* ASVs per specimen than KTx patients (*P* = 0.04) and HC participants (*P* = 0.05) (Figure 6C).

### Clinical Factors, Outcomes, and the Nasal Microbiota

We next evaluated the relationship among the nasal microbiota, clinical factors, and outcomes in the cohort. There were no significant differences in the nasal abundances of the most common genera on the basis of age 65 years or older (Supplemental Table 4). The relative abundance of *Peptoniphilus* was significantly higher in male patients than in female patients (adjusted *P* value <0.10) (Supplemental Table 5). Within the entire cohort, 63 participants received antibiotics within 1 year of nasal swab collection and 28 participants did not. The relative abundance of *Moraxella*, *Petoniphilus*, and *Cutibacterium* was significantly lower in the participants who received antibiotics within 1 year than in the participants who did not (adjusted *P* value <0.10) (Supplemental Table 6). Among the KTx cohort, 12 KTx recipients were on TMP-SMX prophylaxis for *Pneumocystic jirovecii* prophylaxis and 35 were not. There were no significant differences in the nasal abundance of the most common genera between the KTx recipients on TMP-SMX and those who were not (Supplemental Table 7). In the PD cohort, six PD patients concurrently had *Staphylococcus* peritonitis or developed future *Staphylococcus* peritonitis within 10–12 months (last follow-up) (Staph Peritonitis Group) and 26 PD patients did not (No Staph Peritonitis Group). The nasal abundance of *Staphylococcus* was higher in the Staph Peritonitis Group than in the No Staph Peritonitis Group, but the difference was not statistically significant (median abundance 52% versus 24%, respectively, adjusted *P* value 0.73). There were no significant differences in the nasal abundance of the other most common genera between the *Staph* Peritonitis Group and the No *Staph* Peritonitis Group (Supplemental Table 8). In the PD cohort, ten PD patients had a history of peritonitis and 22 PD patients did not. The relative abundance of *Finegoldia* was significantly lower in the PD patients who had a history of peritonitis than in the PD patients who did not (adjusted *P* value <0.10) (Supplemental Table 9). PD patients who had a history of peritonitis had similar Shannon diversity index and Chao1 index compared with PD patients who did not have a history of peritonitis (Shannon diversity index, median 1.7 versus 2.1, *P* = 0.86; Chao1 index, median 29.5 versus 23.5, *P* = 0.67).

## Discussion

This study aimed to describe the anterior nasal microbiota across different groups of patients with kidney disease. We detect a distinct microbial signature in the anterior nares of PD patients compared with KTx recipients and HC participants.

Many of the most common genera in the kidney cohort overlap with those reported in healthy individuals and include *Staphylococccus*, *Corynebacterium*, *Finegoldia*, and *Cutibacterium*.^15,16^ However, there were some distinct differences among the groups. PD patients had a higher nasal abundance of *Streptococcus* than HC participants or KTx recipients. Interestingly, having a higher nasal abundance of *Streptococcus* has been associated with respiratory infections, such as bronchiolitis, in infants.^17^ Although the most common type of infectious peritonitis is *Staphylococcus* in origin, *Streptococcus* peritonitis also occurs in PD patients. Our study was not able to directly address whether PD patients with nasal abundance of *Streptococcus* are associated with *Streptococcus* peritonitis and/or respiratory viral infections.

In our analysis, we noticed a higher nasal microbial diversity in the PD patients compared with KTx recipients and HC participants. Further analysis showed that part of this increased microbial diversity may be due to a more diverse representation of *Staphylococcus* and *Streptococcus* in PD patients (Figure 6). This may have interesting implications as prior data suggest that PD patients with *S. aureus* colonization had a higher incidence of exit-site infections.^7^ In our study, we did find an increased nasal abundance of *Staphylococcus* in PD patients who had a history of *Staphylococcus* peritonitis and/or developed *Staphylococcus* peritonitis. Although the association was not significant, it could be due to the low number of PD patients in the PD Staph peritonitis group as our study was not powered to detect the differences. At the 16S rRNA level, we were not able to determine *Staphylococcus* at the species level and this limitation prevented us to assess this association in more detail. It is possible that the relative abundance of *Staphylococcus* rather than the presence/absence of *Staphylococcus* in the anterior nares is associated with *Staphylococcus* peritonitis. Future studies using mupirocin to decrease the relative abundance of *Staphylococcus* in the anterior nares and possibly decrease the rates of peritonitis may be informative.

Our data also highlight a strong inverse association between the nasal abundance of *Staphylococcus* and that of *Corynebacterium.* Interesting mechanistic studies have shown a complicated relationship between these two taxa, which represent the most common taxa in the nasal microbiota. One study found that *Corynebacterium* species can secrete antimicrobial peptides against *S. aureus*^18^*.* Another study has shown that *Corynebacterium* species can decrease the virulence of *S. aureus*.^19^ Taken together, our data are consistent with the inverse relationship and suggest potential novel approaches to manipulate the nasal microbiota. For example, because *Staphylococcus* peritonitis is much more common than *Corynebacterium* peritonitis, establishing a *Corynebacterium* dominant nasal microbiota may be preventative of *Staphylococcus* in the nasal passages and possibly decrease the risk for *Staphylococcus* exit-site infection and/or peritonitis.

A surprising result is that we did not find an association between TMP/SMX and nasal microbiota differences. TMP/SMX has broad coverage against gram-positive cocci, including *Staphylococcus* species*.* There are few studies which have investigated the role of oral antibiotics on the nasal microbiota, and it is possible that intranasal antibiotics rather than oral antibiotics may more efficiently affect the nasal microbiota. Although our study is limited by the population size and the cross-sectional nature, our study raises this possibility.

There are several limitations to our study. As mentioned earlier, we are unable to assess species-level identification *via* 16S rRNA gene sequencing of the V4–V5 hypervariable region. Future studies using whole gene 16S rRNA gene sequencing or metagenomic sequencing may provide better resolution on the intricate intraspecies competition between the microbiota, particularly between *Staphylococcus* species and *Corynebacterium* species*.* Given the low biomass of the nasal microbiota, environmental contamination and/or contamination through the DNA processing steps could artificially introduce microbiota in our specimens. However, we did sequence negative controls (Figure 3), and the most abundant microbiota identified were not the most common nasal microbiota flora previously reported, suggesting that the nasal microbiota identified in our cohort was present in higher quantities and distinct. An important limitation is that demographics, cultural, and dietary practices could affect the nasal microbiota, and our study is not powered to detect the effect of these potential confounders, so a future larger study needs to be performed to validate our findings. Another important limitation is the cross-sectional design of the nasal microbiota component of the study. Although the cross-sectional nature of our study provides a snapshot of the microbiota across different groups of patients with kidney disease, it does not provide longitudinal changes. Such a longitudinal study may provide better insight into the relationship between the microbiota and outcomes in the populations.

In conclusion, we provide the first description of a distinct nasal microbiota signature in PD patients compared with KTx recipients and HC participants. We find a higher abundance of *Streptococcus* and a more diverse representation of *Staphylococcus* and *Streptococcus* in PD patients. Given the potential relationship between the nasal bacteria and infectious complications in PD patients, further studies are needed to define the nasal microbiota associated with these infectious complications and to conduct studies on the manipulation of the nasal microbiota to prevent such complications.

## Supplementary Material

SUPPLEMENTARY MATERIAL

## Data Availability

Anonymized data created for the study are or will be available in a persistent repository on publication. Raw Data/Source Data; The Database of Genotypes and Phenotypes (dbGaP); Sequencing data that support the findings of this study will be made available in the database of Genotypes and Phenotypes (dbGaP) phs002251.v1.p1 after peer-reviewed acceptance. Partial restrictions to the data and/or materials apply. Local institutional review board approval will be needed to access the data.

## Supplemental Material

Supplemental Table 1. DESeq2 analysis of the nasal microbiota between the PD group and the HC group.

Supplemental Table 2. DESeq2 analysis of the nasal microbiota between the PD group and the KTx group.

Supplemental Table 3. DESeq2 analysis of the nasal microbiota between the KTx group and the HC group.

Supplemental Table 4. Comparison of nasal microbiota on the basis of patient's age at the genus level.

Supplemental Table 5. Comparison of nasal microbiota on the basis of patient's sex at the genus level.

Supplemental Table 6. Comparison of nasal microbiota on the basis of antibiotic usage within the past year at the genus level.

Supplemental Table 7. Comparison of nasal microbiota on the basis of trimethoprim/sulfamethoxazole usage in the kidney transplant recipients at the genus level.

Supplemental Table 8. Comparison of nasal microbiota on the basis of PD patients who developed *Staphylococcus* peritonitis status and PD patients who did not develop *Staphylococcus* peritonitis at the genus level.

Supplemental Table 9. Comparison of nasal microbiota on the basis of PD patients who had a history of peritonitis and PD patients who did not have a history of peritonitis at the genus level.

## References

[1] The Human Microbiome Project Consortium. Structure, function and diversity of the healthy human microbiome. Nature. 2012;486(7402):207–214. doi:10.1038/nature11234 22699609 PMC3564958

[2] Chen F Gao W Yu C, . Age-associated changes of nasal bacterial microbiome in patients with chronic rhinosinusitis. Front Cell Infect Microbiol. 2022;12:786481. doi:10.3389/fcimb.2022.786481 35252024 PMC8891534

[3] Hsiao CJ Paulson JN Singh S, . Nasal microbiota and infectious complications after elective surgical procedures. JAMA Netw Open. 2021;4(4):e218386. doi:10.1001/jamanetworkopen.2021.8386 33914049 PMC8085724

[4] Luzar MA Coles GA Faller B, . Staphylococcus aureus nasal carriage and infection in patients on continuous ambulatory peritoneal dialysis. N Engl J Med. 1990;322(8):505–509. doi:10.1056/NEJM199002223220804 2300122

[5] Nouwen JL Fieren MW Snijders S Verbrugh HA van Belkum A. Persistent (not intermittent) nasal carriage of Staphylococcus aureus is the determinant of CPD-related infections. Kidney Int. 2005;67(3):1084–1092. doi:10.1111/j.1523-1755.2005.00174.x 15698449

[6] Ong LM Ch'ng CC Wee HC, . Risk of peritoneal dialysis-related peritonitis in a multi-racial Asian population. Perit Dial Int. 2017;37(1):35–43. doi:10.3747/pdi.2015.00141 27147287

[7] Mupirocin Study Group. Nasal mupirocin prevents Staphylococcus aureus exit-site infection during peritoneal dialysis. J Am Soc Nephrol. 1996;7(11):2403–2408. doi:10.1681/ASN.V7112403 8959632

[8] Szeto CC Li PK Johnson DW, . ISPD catheter-related infection recommendations: 2017 update. Perit Dial Int. 2017;37(2):141–154. doi:10.3747/pdi.2016.00120 28360365

[9] Di Tommaso P Chatzou M Floden EW Barja PP Palumbo E Notredame C. Nextflow enables reproducible computational workflows. Nat Biotechnol. 2017;35(4):316–319. doi:10.1038/nbt.3820 28398311

[10] Ewels PA Peltzer A Fillinger S, . The nf-core framework for community-curated bioinformatics pipelines. Nat Biotechnol. 2020;38(3):276–278. doi:10.1038/s41587-020-0439-x 32055031

[11] Straub D Blackwell N Langarica-Fuentes A Peltzer A Nahnsen S Kleindienst S. Interpretations of environmental microbial community studies are biased by the selected 16S rRNA (gene) amplicon sequencing pipeline. Front Microbiol. 2020;11:550420. doi:10.3389/fmicb.2020.550420 33193131 PMC7645116

[12] Martin M. Cutadapt removes adapter sequences from high-throughput sequencing reads. EMBnet J. 2011;17(1):10. doi:10.14806/ej.14817.14801.14200

[13] Callahan BJ McMurdie PJ Rosen MJ Han AW Johnson AJ Holmes SP. DADA2: high-resolution sample inference from Illumina amplicon data. Nat Methods. 2016;13(7):581–583. doi:10.1038/nmeth.3869 27214047 PMC4927377

[14] Quast C Pruesse E Yilmaz P, . The SILVA ribosomal RNA gene database project: improved data processing and web-based tools. Nucleic Acids Res. 2012;41(D1):D590–D596. doi:10.1093/nar/gks1219 23193283 PMC3531112

[15] Oh J Conlan S Polley EC Segre JA Kong HH. Shifts in human skin and nares microbiota of healthy children and adults. Genome Med. 2012;4(10):77. doi:10.1186/gm378 23050952 PMC3580446

[16] Pereira PAB Aho VTE Paulin L Pekkonen E Auvinen P Scheperjans F. Oral and nasal microbiota in Parkinson's disease. Parkinsonism Relat Disord. 2017;38:61–67. doi:10.1016/j.parkreldis.2017.02.026 28259623

[17] Hasegawa K Linnemann RW Mansbach JM, . Nasal airway microbiota profile and severe bronchiolitis in infants: a case-control study. Pediatr Infect Dis J. 2017;36(11):1044–1051. doi:10.1097/INF.0000000000001500 28005692 PMC5479744

[18] Menberu MA Liu S Cooksley C, . Corynebacterium accolens has antimicrobial activity against Staphylococcus aureus and methicillin-resistant *Staphylococcus aureus* pathogens isolated from the sinonasal niche of chronic rhinosinusitis patients. Pathogens. 2021;10(2):207. doi:10.3390/pathogens10020207 33672855 PMC7918835

[19] Ramsey MM Freire MO Gabrilska RA Rumbaugh KP Lemon KP. Staphylococcus aureus shifts toward commensalism in response to Corynebacterium species. Front Microbiol. 2016;7:1230. doi:10.3389/fmicb.2016.01230 27582729 PMC4988121

