# Supplementary material for: A Distinct Nasal Microbiota Signature in Peritoneal Dialysis Patients
Source: Kidney360. 2023 Aug 29;4(10):1419–29. doi: 10.34067/KID.0000000000000249 (PMC10615377; doi:10.34067/KID.0000000000000249)
Supplement: SUPPLEMENTARY MATERIAL [file kidney360-4-01419-s001.pdf]

Supplementary Materials

A Distinct Nasal Microbiota Signature in Peritoneal Dialysis Patients

Iman Khan[1], Sylvia Wu[1], Anika Hudson[1], Clayton Hughes[1], Gabriel Stryjniak[1], Lars F. Westblade[2,3], Michael J. Satlin[2,3], Nicholas Tedrow[1], Anne-Catrin Uhlemann[4], Colleen Kraft[5], Darshana M. Dadhania[1,6], Jeffrey Silberzweig[1,7], Iwijn De Vlaminck[8], Carol Li[1], Vesh Srivatana[1,7*], John Richard Lee[1,6*]

1. Division of Nephrology and Hypertension, Department of Medicine, Weill Cornell Medicine, New York, NY, USA
2. Department of Pathology and Laboratory Medicine, Weill Cornell Medicine, New York, NY, USA
3. Division of Infectious Diseases, Department of Medicine, Weill Cornell Medicine, New York, NY, USA
4. Division of Infectious Diseases, Department of Medicine, Vagelos College of Physicians and Surgeons Columbia University, New York, NY
5. Division of Infectious Diseases, Department of Medicine, Emory University School of Medicine, New York, NY
6. Department of Transplantation Medicine, New York Presbyterian Hospital–Weill Cornell Medical Center, New York, NY, USA
7. The Rogosin Institute, New York, NY, USA
8. Department of Biomedical Engineering, Cornell University, Ithaca, NY, USA

* V.S. and J.R.L. contributed equally to this work. Correspondence to Dr. Vesh Srivatana, ves2009@nyp.org, or Dr. John R. Lee, jrl2002@med.cornell.edu

Table of Contents

Page 3	SI Table 1
Page 4	SI Table 2
Page 5	SI Table 3
Page 6	SI Table 4
Page 7	SI Table 5
Page 8	SI Table 6
Page 9	SI Table 7
Page 10	SI Table 8
Page 11	SI Table 9

SI Table 1

Genus	ASV	Base Mean	log2 Fold Change	Standard error	Wald stat	P value	Adj P value
Finegoldia ASV #1	1f016ec26e4774c86f029301b556de28	24.3	-26.4	2.9	-9.0	3.4E-19	1.4E-17
Anaerococcus ASV #1	ae7cf0f32080cf5e62b3fbd903a997af	16.5	-25.9	3.0	-8.7	2.2E-18	4.4E-17
Lawsonella ASV #1	b89dbfe5c74375ff3d4922b41c470740	49.0	-7.3	2.9	-2.5	1.3E-02	5.1E-02
Corynebacterium ASV #2	c91b64f1467982a81922d331a310f5dc	1172.2	-3.7	1.6	-2.2	2.5E-02	9.2E-02
Bacillus ASV #1	5565e52f91bc244013249656795409ca	18.7	6.9	1.8	3.8	1.7E-04	7.7E-04
Staphylococcus ASV #2	b273d7d05f0525bb87418b0138d90605	86.0	11.8	2.2	5.3	1.2E-07	6.1E-07
Porphyromonas ASV #1	969732ae717bf5a9282f4781499aadb4	10.0	21.4	3.0	7.2	6.3E-13	3.6E-12
Abiotrophia ASV #1	afce1dc2c06acb90ad5d3da0fffbcd02	9.7	22.3	2.8	7.9	3.5E-15	2.4E-14
Streptococcus ASV #1	4481bc66d3a54f79e6abb8b557a2a104	19.3	23.2	2.9	7.9	2.5E-15	2.4E-14
Staphylococcus ASV #1	7b06c22a4ff85c9b2cbbf57a53462e13	41.9	23.4	3.0	7.9	3.6E-15	2.4E-14
Corynebacterium ASV #1	0560198302010f9b110d4ec9897e14d0	45.9	24.5	3.0	8.2	1.8E-16	2.3E-15

DESeq2 Analysis of the Nasal Microbiota between the PD Group and the HC Group. The listed amplicon sequence variant (ASV) were determined to be significantly different using an adjusted p value of 0.10. Base Mean, mean of normalized counts for all samples. Wald stat, Wald statistic. Positive log2 Fold Change is higher in the PD Group.

SI Table 2

Genus	ASV	Base Mean	log2 Fold Change	Standard error	Wald stat	P value	Adj P value
Staphylococcus ASV #3	0f89ba63b47f3b624c13293cb4d56486	16.5	-25.6	2.9	-8.8	1.9E-18	2.0E-16
Porphyromonas ASV #1	969732ae717bf5a9282f4781499aadb4	7.4	21.6	2.9	7.4	1.1E-13	4.8E-12
Abiotrophia ASV #1	afce1dc2c06acb90ad5d3da0fffbcd02	7.3	23.3	2.8	8.4	4.3E-17	3.0E-15
Corynebacterium ASV #3	36f00e24a741c19a5ee20f58919004ad	12.4	24.0	2.9	8.2	1.7E-16	8.8E-15
Staphylococcus ASV #2	b273d7d05f0525bb87418b0138d90605	65.8	24.8	2.4	10.2	1.5E-24	3.1E-22

DESeq2 Analysis of the Nasal Microbiota between the PD Group and the KTx Group. The listed amplicon sequence variant (ASV) were determined to be significantly different using an adjusted p value of 0.10. Base Mean, mean of normalized counts for all samples. Wald stat, Wald statistic. Positive log2 Fold Change is higher in the PD Group.

SI Table 3

Genus	ASV	Base Mean	log2 Fold Change	Standard error	Wald stat	P value	Adj P value
Anaerococcus ASV #1	ae7cf0f32080cf5e62b3fbd903a997af	12.0	-25.4	3.0	-8.5	2.8E-17	2.2E-15
Corynebacterium ASV #4	9f6c00ff9e1f455f42f211770d60dec0	11.2	-25.3	3.0	-8.4	3.6E-17	2.2E-15
Staphylococcus ASV #3	0f89ba63b47f3b624c13293cb4d56486	12.5	21.6	3.0	7.2	7.4E-13	2.8E-11
Staphylococcus ASV #1	7b06c22a4ff85c9b2cbbf57a53462e13	11.8	22.9	3.0	7.6	2.8E-14	1.3E-12
Corynebacterium ASV #1	0560198302010f9b110d4ec9897e14d0	237.4	27.1	3.0	9.0	2.2E-19	4.1E-17

DESeq2 Analysis of the Nasal Microbiota between the KTx Group and the HC Group. The listed amplicon sequence variant (ASV) were determined to be significantly different using an adjusted p value of 0.10. Base Mean, mean of normalized counts for all samples. Wald stat, Wald statistic. Positive log2 Fold Change is higher in the KTx Group.

SI Table 4

	Age >=65	Age < 65		
	n = 28	n = 63		
Genus	Median Abundance	Median Abundance	P value	Adj P value
Staphylococcus	0.187	0.346	0.11	0.40
Finegoldia	0.000	0.001	0.11	0.40
Anaerococcus	0.001	0.013	0.14	0.40
Moraxella	0.000	0.000	0.18	0.40
Unspecified Neisseriaceae	0.000	0.000	0.30	0.48
Streptococcus	0.012	0.007	0.32	0.48
Corynebacterium	0.124	0.139	0.56	0.72
Peptoniphilus	0.000	0.000	0.66	0.74
Cutibacterium	0.003	0.002	0.81	0.81

Comparison of Nasal Microbiota Based on Patient's Age at the Genus Level. P value was calculated between groups using Wilcoxon rank sum test. Adjusted p value (Adj P value) was calculated using Benjamini-Hochberg adjustment.

SI Table 5

Genus	Female Sex<br>n = 50<br>Median Abundance	Male Sex<br>n = 41<br>Median Abundance	P value	Adj P value
Peptoniphilus	0.000	0.005	0.01	0.10
Finegoldia	0.000	0.002	0.07	0.31
Anaerococcus	0.002	0.017	0.19	0.51
Corynebacterium	0.101	0.197	0.23	0.51
Cutibacterium	0.002	0.007	0.38	0.64
Moraxella	0.000	0.000	0.43	0.64
Unspecified *Neisseriaceae*	0.000	0.000	0.90	0.91
Streptococcus	0.008	0.010	0.90	0.91
Staphylococcus	0.285	0.250	0.91	0.91

Comparison of Nasal Microbiota Based on Patient's Sex at the Genus Level. P value was calculated between groups using Wilcoxon rank sum test. Adjusted p value (Adj P value) was calculated using Benjamini-Hochberg adjustment.

SI Table 6

Genus	Abx Hx 1 year (n = 63) median abundance	No Abx Hx 1 year (n = 28) median abundance	P value	Adj P Value
Moraxella	0.000	0.000	0.002	0.02
Peptoniphilus	0.000	0.007	0.01	0.04
Cutibacterium	0.000	0.009	0.01	0.04
Streptococcus	0.010	0.003	0.05	0.10
Unspecified *Neisseriaceae*	0.000	0.002	0.06	0.10
Staphylococcus	0.275	0.211	0.07	0.10
Corynebacterium	0.097	0.382	0.08	0.10
Finegoldia	0.000	0.001	0.18	0.20
Anaerococcus	0.006	0.014	0.60	0.60

Comparison of Nasal Microbiota Based on Antibiotic Usage within the Past Year at the Genus Level. P value was calculated between groups using Wilcoxon rank sum test. Adjusted p value (Adj P value) was calculated using Benjamini-Hochberg adjustment. Abx, antibiotic; Hx, history.

SI Table 7

Genus	KTx on TMP/SMX n = 12 Median Abundance	KTx off TMP/SMX n = 25 Median Abundance	P value	Adj P value
Finegoldia	0.000	0.002	0.02	0.15
Peptoniphilus	0.000	0.000	0.04	0.15
Anaerococcus	0.0002	0.007	0.06	0.15
Corynebacterium	0.101	0.314	0.36	0.59
Cutibacterium	0.000	0.000	0.37	0.59
Staphylococcus	0.363	0.487	0.53	0.71
Unspecified *Neisseriaceae*	0.000	0.000	0.70	0.80
Streptococcus	0.002	0.005	0.84	0.84
Moraxella	0.000	0.000	NA	NA

Comparison of Nasal Microbiota Based on Trimethoprim/Sulfamethoxazole usage in the Kidney Transplant Recipients at the Genus Level. P value was calculated between groups using Wilcoxon rank sum test. Adjusted p value (Adj P value) was calculated using Benjamini-Hochberg adjustment. P values with NA were unable to be calculated because the abundances were 0 in both groups. KTx, Kidney Transplant; TMP/SMX, trimethoprim/sulfamethoxazole.

SI Table 8

Genus	PD Staph Future Peritonitis n = 6 Median Abundance	PD No Staph Future Peritonitis n = 26 Median Abundance	P value	Adj P value
Unspecified *Neisseriaceae*	0.000	0.000	0.08	0.66
Staphylococcus	0.521	0.240	0.38	0.73
Streptococcus	0.009	0.035	0.38	0.73
Anaerococcus	0.0002	0.022	0.39	0.73
Peptoniphilus	0.000	0.000	0.48	0.73
Cutibacterium	0.001	0.000	0.55	0.73
Finegoldia	0.000	0.000	0.76	0.87
Corynebacterium	0.101	0.098	1.00	1.00
Moraxella	0.000	0.000	NA	NA

Comparison of Nasal Microbiota Based on PD patients who developed *Staphylococcus* peritonitis status and PD patients who did not develop *Staphylococcus* peritonitis at the Genus Level. P value was calculated between groups using Wilcoxon rank sum test. Adjusted p value (Adj P value) was calculated using Benjamini-Hochberg adjustment. P values with NA were unable to be calculated because the abundances were 0 in both groups. PD Staph peritonitis cohort was defined as PD patients who concurrently had *Staphylococcus* peritonitis or developed *Staphylococcus* peritonitis withn 10 to 12 months (last follow up).

SI Table 9

Genus	PD Patients History of Peritonitis (n = 10) median abundance	PD Patients No History of Peritonitis (n = 22) median abundance	P value	Adj P Value
Finegoldia	0.000	0.001	0.005	0.04
NeisseriaceaeUnspecified	0.000	0.000	0.06	0.24
Peptoniphilus	0.000	0.000	0.10	0.26
Cutibacterium	0.000	0.002	0.14	0.28
Corynebacterium	0.047	0.141	0.32	0.51
Staphylococcus	0.428	0.240	0.48	0.64
Anaerococcus	0.020	0.013	0.76	0.86
Streptococcus	0.018	0.027	0.95	0.95
Moraxella	0.000	0.000	NA	NA

Comparison of Nasal Microbiota Based on PD patients who had a history of peritonitis and PD patients who did not have a history of peritonitis at the Genus Level. P value was calculated between groups using Wilcoxon rank sum test. Adjusted p value (Adj P value) was calculated using Benjamini-Hochberg adjustment. P values with NA were unable to be calculated because the abundances were 0 in both groups.
